# Antiproliferative Effects of Methanolic Root Extracts of Eichhornia crassipes Against a Skin Melanoma Cell Line: An In Vitro Study

**DOI:** 10.7759/cureus.34545

**Published:** 2023-02-02

**Authors:** Noufal K P, Rajesh B, Sujith S Nair

**Affiliations:** 1 Anatomy, Bharath Institute of Higher Education and Research, Chennai, IND; 2 Anatomy, Sri Lakshmi Narayana Institute of Medical Sciences, Pondicherry, IND; 3 Pharmaceutics, Crescent College of Pharmaceutical Sciences, Kannur, IND

**Keywords:** sk-mel-5 cell line, root extract, petiole extract, melanoma, eichhornia crassipes

## Abstract

Background

Melanoma is the most aggressive form of skin cancer, accounting for 3% of all malignant cancers. Phytochemicals and their related compounds are found in various parts of the plant *Eichhornia crassipes* and have a variety of pharmacological actions. The current research was intended to compare and evaluate the anti-proliferative action of methanolic extracts of *E. crassipes* roots and petioles against the Sloan Kettering Melanoma (SK-Mel-5) cell line.

Materials and methods

The waters around Ezhikkara, Ernakulum, Kerala, were discovered to contain* E. crassipes*. We used a Soxhlet extractor to get this concentrated liquid. For this test, we employed a methanolic extract of roots and petioles to determine the extent to which different concentrations of the extract inhibited cell proliferation. Data on absorbance were reported as a mean standard deviation. Using Probit analysis, the IC_50_ was calculated by evaluating the gradient of the regression line to get a value.

Results

Concentrations of methanolic root and petiole extracts of 12.5 µg/ml, 25 µg/ml, 50 µg/ml, 100 µg/ml, and 200 µg/ml were analyzed. The methanol petiole extract reduced the viability of SK-Mel-5 cells more than the root extract, with IC_50_ values of 323.59 µg/ml and 174.70 µg/ml of the test sample concentration, respectively. The regression equation for the root extract was y = -0.1264x + 90.902 and R^2^ = 0.845, and for the petiole extract, it was y = -0.2187x + 88.206 and R^2^ = 0.917.

Conclusion

The current study found that increasing the concentration of methanolic extracts of roots and petioles of *E. crassipes *exhibited an increased cell growth inhibition rate. However, methanolic petiole extracts were more cytotoxic than the roots. Thus, the current study demonstrated the therapeutic use of *E. crassipes* as an anticancer agent, thereby providing a valuable alternative for enabling the early management of melanoma.

## Introduction

The number of people diagnosed with cancer is anticipated to reach 21 million by 2030, making it one of the top causes of mortality and morbidity globally [1]. Because plants play so many important biological roles, such as antioxidant, anti-inflammatory, and anticancer effects, medicines derived from plants are seen as potential agents for treating a wide range of diseases. Approximately 80% of the global population is reliant on traditional medicinal products, and approximately 60% of anti-cancer medications on the market or in clinical studies are naturally derived [1,2]. With a better knowledge of the benefits of such traditional remedies, the implications against various kinds of cancers have also been outlined [3]. One such plant is *Eichhornia crassipes* belonging to the Pontederiaceae family, which provides new avenues of biomolecules that can be utilized or streamlined against cancer. *E. crassipes* is a widespread waterweed commonly referred to as water hyacinth that can be found in abundance floating over the surface or indeed explicitly rooted into the water bodies [4].

Flowers, fruits, seeds, roots, rhizomes, stems, leaves, and bark all contain substances with varying pharmacological effects; these are called phytochemicals. Many primary and secondary metabolic products, including alkaloids, flavonoids, lignans, saponins, terpenes, taxanes, vitamins, minerals, glycosides, oils, bioactive compounds, and others, play important roles in inhibiting peptides, enzymes, and signaling cascades that activate cancer cells [1]. The protective efficacy against reactive oxygen species and free radicals is greater with natural antioxidants including polyphenols, carotenoids, and flavonoids due to their increased bioavailability. Chemical components that acted synergistically in the crude extract and allowed for its maximum activities were identified using spectroscopy of the separated ingredients [5].

About 3% of all malignant carcinomas are melanomas, making them the worst type of skin cancer. As a consequence of its high propensity for metastasis and invasion, it accounts for over 75% of the total skin cancer burden worldwide. In the United States, it ranks fifth among male cancers and seventh among female cancers, and in India, it is sixth among the most common cancers majorly occurring in females [6]. Scientists are becoming increasingly interested in screening an increasing variety of vegetative species in order to detect plant-based compounds that could be used to prevent or treat cancer-reported incidents [4]. *E. crassipes* is said to have a plethora of health benefits, which include nutrients, minerals, anti-microbial action, antioxidant properties, and cytotoxic activity [7-10]. Earlier studies have demonstrated that several phytochemical constituents from the leaves of *E. crassipes* possess anti-carcinogenic propensity [11-13]. Concentrations of heavy metals such as aluminum, barium, cobalt, copper, cadmium, chromium, iron, lead, nickel, manganese, and zinc were found to be greater in roots. Water hyacinth shoots had higher levels of nutrients such as ammonia, nitrate, nitrite, and phosphate [9,10].

According to research, the elemental composition of the roots might not be identical to that of the leaves for vegetation types owing to variations in the rates and effects of metabolic activities, as well as the impact of the immediate geographic environments [4]. Phytochemical findings indicate that mutation rates in the plants result in differences in chemical compositions in distinct regions [14]. This prompts related studies on various plant parts to investigate the phytochemical profile of the plants extensively. Therefore, the goal of this study was to examine the efficacy of methanolic extracts of *E. crassipes*' roots and petioles in inhibiting the proliferation of the Sloan Kettering Melanoma (SK-Mel-5) cell line.

## Materials and methods

Plant collection, identification, and extract preparation

The waters of Ezhikkara, Ernakulum, Kerala, were dredged out of roots and shoots of water hyacinth. Affirmation of the collected plant's identity was done which included leaves that are well above the water's surface on stalks and are thick, waxy, spherical, and shiny. The leaves have sides that are often undulating, gently incurved, and widely ovate to round, measuring 10-20 cm in diameter. Leaf veins are long, narrow, numerous, and thick. Leaf stalks are spongy and bulbous. A single spike of 8-15 beautiful blooms is carried at the summit of the 50 cm-long, upright stalk. The flowers feature six petals that range in color from purplish blue to lavender to pinkish, with a golden center and blue borders. The extract preparation was performed at Sri Lakshmi Narayana Institute of Medical Sciences, Puducherry. The flowers were thoroughly rinsed and finely ground in a laboratory blender into homogeneous particulate. The 10-gram air-dried ground specimen was extracted with methanol solvent in steps. After filtering root and petiole extracts and evaporating solvents in a rotary evaporator at 40° to 45°C, the extract residues were weighed, and various concentrations of each extract were prepared. The extracts were then dried and kept in a fridge for subsequent use under reduced pressure. A preliminary phytochemical analysis of *E. crassipes* root and petiole extracts was carried out to identify various plant components such as alkaloids, flavonoids, polyphenolic compounds, tannins, saponins, terpenoids, anthocyanins, and polypeptides. The extracts of the roots and petioles were then chosen for the 3-[4,5-dimethylthiazol-2-yl]2,5-diphenyltetrazolium bromide (MTT) assay, which was used to determine the rate of inhibitory effects at different concentration levels.

Cell line and culture media maintenance

The SK-Mel-5 human malignant melanoma cell line was offered for sale by the National Centre for Cell Sciences (NCCS), which is based in Pune, India. All of the isolates were cultured in Dulbecco's modified Eagle's medium (DMEM; HIMEDIA, Thane, Maharashtra, India) supplemented with 10% heat-inactivated fetal bovine serum (FBS) and 1% each of penicillin (100U/ml), streptomycin (100g/ml), and amphotericin B (2.5g/ml). Cells were cultured in TC flasks in a humidified CO_2_ incubator at 37 degrees Celsius (25 cm^2^). Cells were kept in a low-passage stage in a liquid nitrogen vapor environment with 20% FBS and 10% dimethylsulfoxide added to the cell culture medium (DMSO).

MTT assay

In 96-well microtiter plates with DMEM medium, the SK-MEL-5 cell line was implanted at 5 x 103 cells/ml. The cells were incubated overnight for adherence. The cells were cultured in three replicates with *E. crassipes* roots and petioles methanol extract at various doses (12.5 µg/ml, 25 µg/ml, 50 µg/ml, 100 µg/ml, 200 µg/ml), and cells were cultured for 72 hours. Following that, the cells were treated with MTT at a dosage of 2 g/ml. The samples were kept at 37ᵒC for three hours before adding DMSO to each well and measuring the absorption spectrum at 492 nm with a microplate reader.

Images were taken at regular intervals over the course of 48 hours using an inverted phase contrast tissue culture microscope on both the treatment and control wells (Labomed TCM-400, Labo America Inc, Fremont CA, USA with a MICAPS HD camera). Any observable morphological alterations were used to calculate cytotoxicity.

The percentage of cell growth inhibition or the percentage of cytotoxicity was computed by applying the equation:

Percentage of cell viability = (Average absorbance of treated / Average absorbance of control) x 100.

Statistical analysis

The statistical analyses were performed using IBM SPSS Statistics for Windows, Version 26 (Released 2019; IBM Corp., Armonk, New York, United States). The absorbance readings were signified as Mean ± SD, and the estimates of cell growth inhibition were calculated. The IC_50_ was deduced using the slope of the regression equation, y = mx + c, and was ascertained using Probit analysis.

## Results

The cytotoxicity of methanol extracts of *E. crassipes* roots and petioles was compared against the SK-Mel-5 cell line (Table 1).

**Table 1:** Probit analysis IC50 of the methanolic root and petiole extracts of E. crassipes against the SK-Mel-5 cell line PE: Petiole extract; RE: root extract; SK-Mel-5: Sloan Kettering Melanoma

					Drug concentration (µg/ml) cell line: SK-Mel-5									
Parameter	Blank		Untreated		12.5 µg/ml		25 µg/ml		50 µg/ml		100 µg/ml		200 µg/ml	
	PE	RE	PE	RE	PE	RE	PE	RE	PE	RE	PE	RE	PE	RE
Abs reading 1	0.06	0.05	1.27	1.28	1.19	1.21	1.07	1.13	0.95	1.04	0.80	0.951	0.65	0.89
Abs reading 2	0.05	0.05	1.30	1.29	1.18	1.21	1.08	1.13	0.95	1.05	0.81	0.96	0.63	0.90
Abs reading 3	0.05	0.05	1.28	1.28	1.20	1.22	1.07	1.14	0.95	1.06	0.80	0.95	0.625	0.90
Mean abs±SD	0.05± 0.01	0.05 ± 0.01	1.28± 0.01	1.28± 0.01	1.18± 0.01	1.21 ± 0.01	1.07± 0.01	1.13 ± 0.01	0.95± 0.01	1.05 ± 0.01	0.80± 0.002	0.954 ± 0.01	0.63± 0.01	0.90 ± 0.01
Mean abs (Sample-Blank)	0	0	1.23	1.23	1.13	1.16	1.02	1.10	0.90	1.00	0.75	0.91	0.58	0.85
Cell viability (%)	0	0	100	100	91.96	94.32	82.62	88.00	72.79	81.27	61.09	73.40	47.03	68.53
Cell growth inhibition (%)	0	0	0	0	8.04	5.68	17.38	12	27.21	18.73	38.91	26.6	52.97	31.47

Concentration levels of methanol extract of 12.5 µg/ml, 25 µg/ml, 50 µg/ml, 100 µg/ml, and 200 µg/ml were tested. The current study found that methanol extracts of roots and petioles reduced the survivability of tumor cell lines, with the petiole extract having the greatest effect. Cell growth inhibition was enhanced as concentration was increased. 

**Figure 1:**
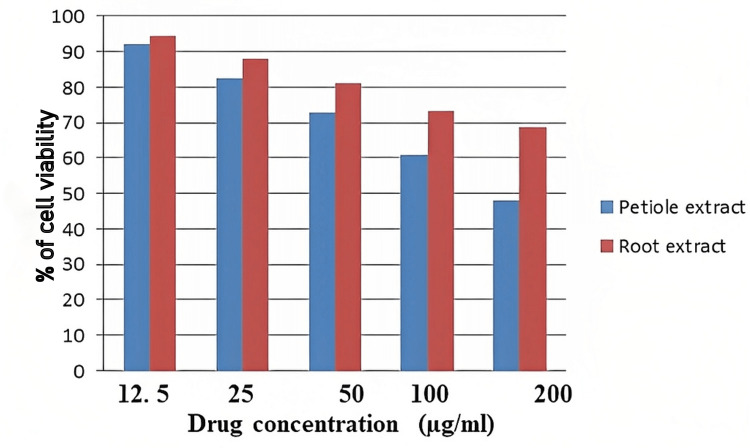
Anticancer activity of methanolic root and petiole extracts of E. crassipes against the SK-Mel-5 cell line SK-Mel-5: Sloan Kettering Melanoma

In accordance with the study results, the methanolic extract of *E. crassipes* petioles had an increased cytotoxic influence on the cell lines than the root extract. As shown in Figure 1, the extract demonstrated a significant concentration-dependent percentage of cell viability.

The administration of varying amounts of *E. crassispes* methanolic root and petiole extracts resulted in a dose-dependent decrease in the cell viability of SK-Mel-5 cells. Cell viability percentages at 200 µg/ml for root and petiole extracts were reported to be 68.53% and 47.03%, respectively.

**Figure 2:**
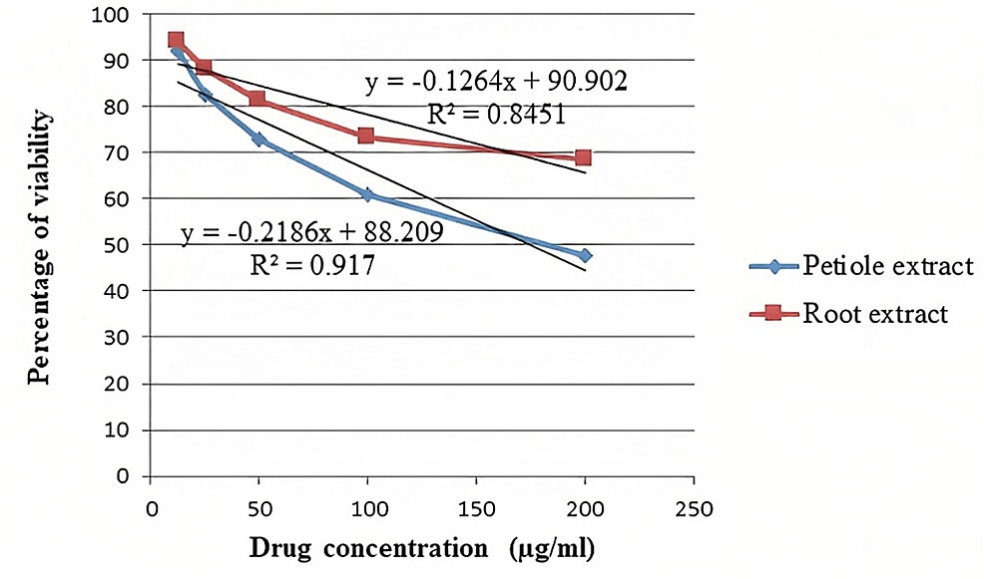
Dose-response curve of methanolic root and petiole extracts of E. crassipes against the SK-Mel-5 cell line SK-Mel-5: Sloan Kettering Melanoma

Figure 2 shows the effect of increasing doses of *E. crassispes* extracts on SK-Mel-5 cell proliferation.

Root and petiole methanolic extracts of *E. crassipes* were reported to be cytotoxic against tumor cell lines, with IC_50_ values of 323.59 g/ml and 174.70 g/ml, respectively. The regression equation for the root extract was y = -0.1264x + 90.902 with R2 = 0.845, and for the petiole extract, it was y = -0.2187x + 88.206 with R2 = 0.917.

## Discussion

Anticancer, antioxidant, anti-inflammatory, skin-lightening, neuroprotective, and hepatoprotective activities are only some of the pharmacological benefits associated with *E. crassipes* extracts. Allelopathic, insecticidal, and antibacterial activities are a few further examples of the documented biological impacts [15]. Several previous studies have documented the characterization of biomolecules in *E. crassipes* leaf extracts [7,16,17]. However, there is a scarcity of studies that report the anticarcinogenic prospects of the plant's root and petiole extracts [18]. The current study shows that the petiole extract of *E. crassipes* does indeed have a higher percentage of cell inhibition than the root extract.

Localized melanoma can be treated surgically with appropriate safety limits. Metastatic melanoma, on the other hand, has a poor prognosis. There are multiple therapeutic strategies currently available that seek to prevent malignant cells from migrating invasively. When cancer is diagnosed at a late stage, most chemotherapies fail to produce the desired biological responses and are instead linked to an increase in cell resistance. Since complexes recovered from these phytochemicals have shown anti-inflammatory, triggering apoptosis, and antitumorigenic characteristics, interest in natural products for cancer prevention and treatment has skyrocketed. Some preclinical and clinical research has attempted to exemplify the beneficial effects of plant biomolecules and derivative products in the treatment of melanoma [19]. In a prior study, methanolic leaf extracts were tested against B16F10 melanoma tumor cells, and the tumor volume regression was noticed in hybrid mouse models when contrasted with controls [20].

Malignant melanoma is a multifactorial disease, with exorbitant ultraviolet radiation exposure being the primary cause of its development. Melanoma patients may benefit from using plant extracts in treatment because they slow tumor growth, reduce metastasis risk, and promote regression of tumor angiogenesis. Multiple metabolic pathways contribute to the inhibition of tumor responses. This leads to a decrease in haphazard cell division, apoptosis, tissue invasion, and inflammation [19], as signaling carcinogenic pathways are regulated. Flavonoid-rich herbs have been used in traditional medicine for centuries because of their ability to boost the immune system via their antioxidant, anti-inflammatory, anti-allergenic, and antithrombotic pharmacological qualities. Certain research has shown that flavonoids have greater antioxidant action. Flavonoids act as photoreceptors, assist guard against invading infections, protect against harmful UV-B rays, and may even reduce oxidative stress [21].

A previous study found tannins, phlobatannin, steroids, terpenoids, alkaloids, flavonoids, phenolic contents, anthraquinone, and cardiac glycosides in sequential extracts of water hyacinth shoot and roots [22]. However, phlobatannin and cardiac glycosides were not identified in the root system. According to Gonzalez et al., the optimized potency of a medicinal herb could not be attributed to a single primary ingredient, but rather to the cumulative action of various biomolecules in the plant [23]. Mtewa et al. reported that benzene-1,4-diol and nonanedioic acid were separated from the leaves and roots of *E. crassipes*, respectively [18]. While conventional anticancer drugs indicated 50% to 62% cytotoxic effects at different samples from various test cell lines, Parveen et al. proved that an aqueous fraction of *E. crassipes* leaf expressed 51% and 44% cytotoxic propensity against the NCI-H322 cell line, respectively [24].

The larvicidal activity of the ethanol extract of *E. crassipes* leaves and shoot against Culex quinquefasciatus was higher than that of the other solvent extracts, which may be due to the presence of metabolites such anthraquinones, alkaloids, and flavonoids, as reported by Jayanthi et al. [25]. Wound contractility was greatly improved by ointments containing methanol extracts of *E. crassipes* leaves (both 10% and 15% leaves extract) compared to the control [26]. This was likely due to the presence of phenolic chemicals in the leaves. The incorporation of nanoparticles as a drug delivery vehicle to the target tissue is growing in the area of nanotechnology. Some components with anticancer activity may be hampered in drug trials due to the necessity of larger doses [27,28]. The nano-encapsulation of *E. crassipes* extract has the advantage of providing additional biologically active components [29].

Future research will provide insight into the pharmacologically active chemical constituents of the plant and its deterministic effects. This in turn would highlight its potential as a natural, plant-derived cytotoxic activity for cancer therapies.

## Conclusions

The study findings indicate that phytoconstituents in methanolic extracts of *E. crassipes* roots and petioles have efficacious antiproliferative properties in vitro. The cell growth inhibition rate was also noticed to increase as the concentration of the methanolic root and petiole extracts was increased. However, methanolic *E. crassipes* petiole extracts were more cytotoxic than the roots. Thus, the current study demonstrates the therapeutic use of* E. crassipes* as an anticancer agent, thereby providing a valuable alternative for enabling the management of melanoma.
